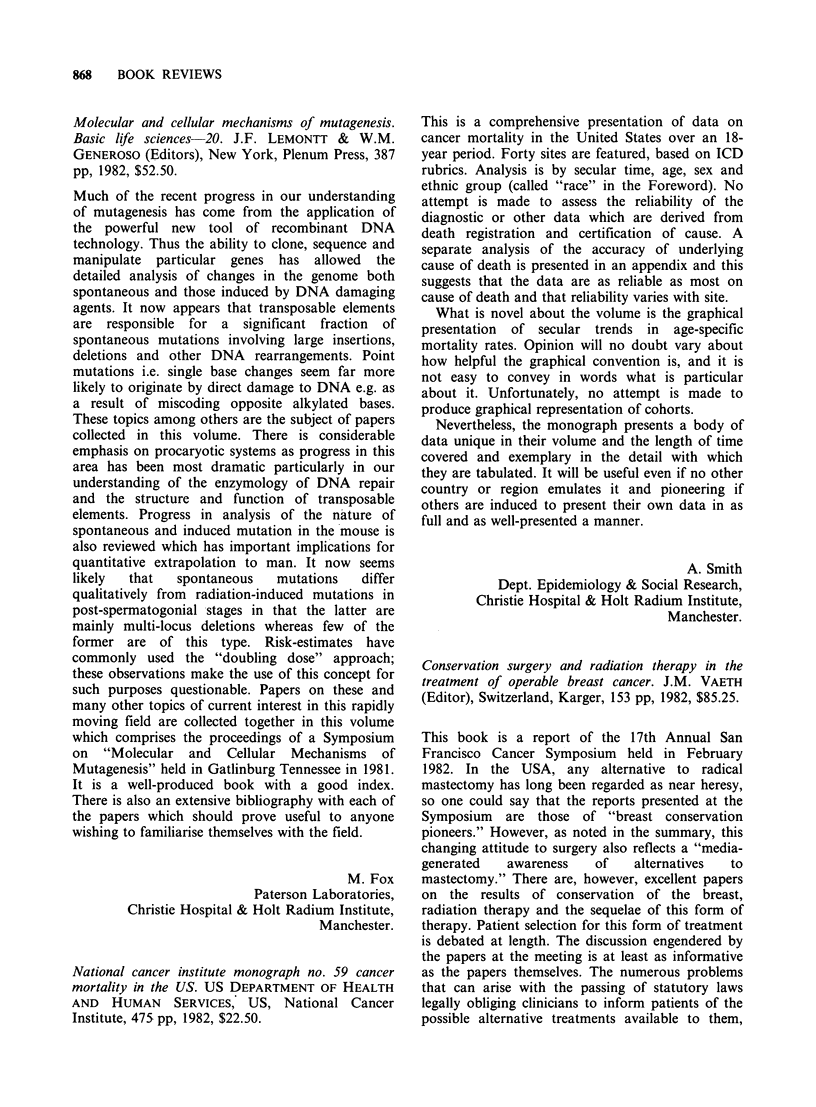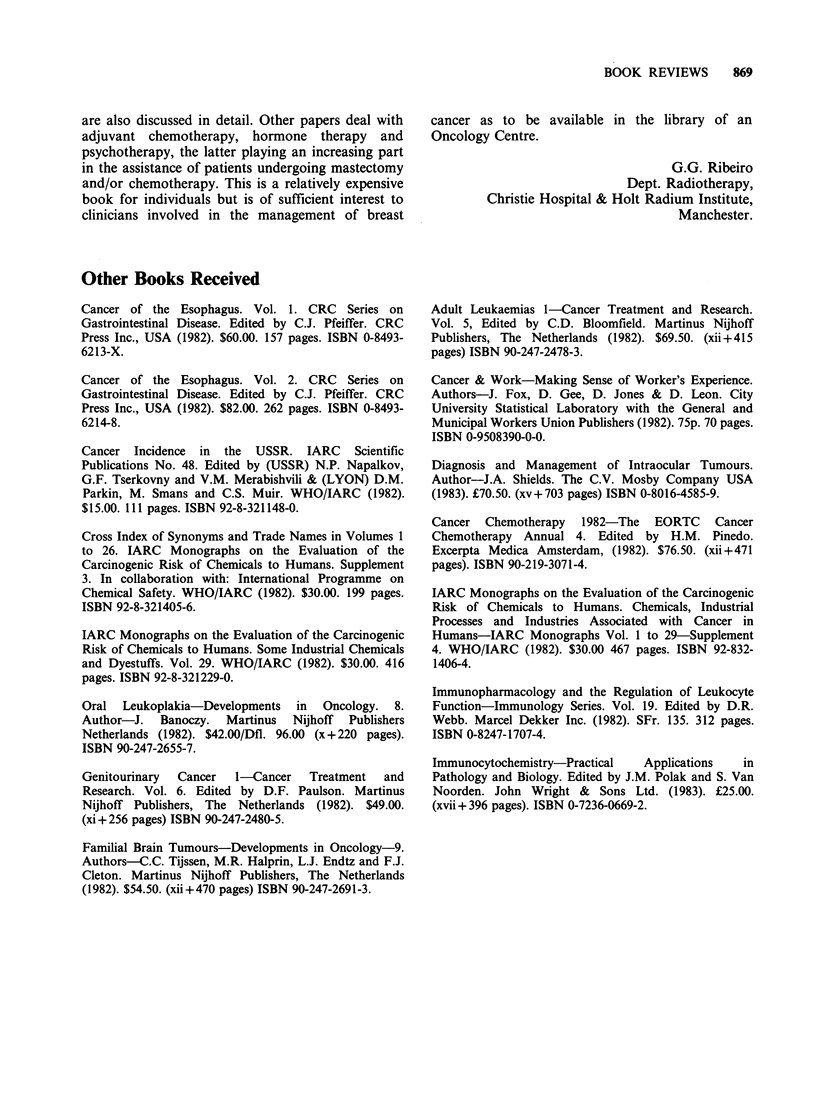# Conservation surgery and radiation therapy in the treatment of operable breast cancer

**Published:** 1983-06

**Authors:** G. G. Ribeiro


Christie Hospital & Holt Radium Institute,

Manchester.

*Conservation surgery and radiation therapy in the treatment of operable breast cancer.* J.M. VAETH (Editor), Switzerland, Karger, 153 pp, 1982, $85.25.

This book is a report of the 17th Annual San Francisco Cancer Symposium held in February 1982. In the USA, any alternative to radical mastectomy has long been regarded as near heresy, so one could say that the reports presented at the Symposium are those of "breast conservation pioneers." However, as noted in the summary, this changing attitude to surgery also reflects a "media-generated awareness of alternatives to mastectomy." There are, however, excellent papers on the results of conservation of the breast, radiation therapy and the sequelae of this form of therapy. Patient selection for this form of treatment is debated at length. The discussion engendered by the papers at the meeting is at least as informative as the papers themselves. The numerous problems that can arise with the passing of statutory laws legally obliging clinicians to inform patients of the possible alternative treatments available to them, are also discussed in detail. Other papers deal with adjuvant chemotherapy, hormone therapy and psychotherapy, the latter playing an increasing part in the assistance of patients undergoing mastectomy and/or chemotherapy. This is a relatively expensive book for individuals but is of sufficient interest to clinicians involved in the management of breast cancer as to be available in the library of an Oncology Centre.

G.G. Ribeiro
Dept. Radiotherapy,
Christie Hospital & Holt Radium Institute,
Manchester.